# High PD-L1 expression on immune cells along with increased density of tumor-infiltrating lymphocytes predicts a favorable survival outcome for patients with loco-regionally advanced head and neck cancer: early results from a prospective study

**DOI:** 10.3389/fonc.2024.1346793

**Published:** 2024-04-04

**Authors:** Tomáš Blažek, Marek Petráš, Pavel Hurník, Petr Matoušek, Lukáš Knybel, Zuzana Zděblová Čermáková, Jan Štembírek, Jakub Cvek, Renata Soumarová

**Affiliations:** ^1^ Department of Oncology, Ostrava University Hospital, Ostrava, Czechia; ^2^ Faculty of Medicine, University of Ostrava, Ostrava, Czechia; ^3^ Third Faculty of Medicine, Charles University, Prague, Czechia; ^4^ Department of Pathology, Ostrava University Hospital, Ostrava, Czechia; ^5^ Department of Otorhinolaryngology, Ostrava University Hospital, Ostrava, Czechia; ^6^ Department of Orofacial Surgery, Ostrava University Hospital, Ostrava, Czechia; ^7^ Department of Oncology, Královské Vinohrady University Hospital, Prague, Czechia

**Keywords:** immune biomarkers, PD-L1 expression, tumor-infiltrating lymphocytes, prognosis, head and neck cancer

## Abstract

**Introduction:**

In the era of personalized medicine and treatment optimization, use of immune biomarkers holds promise for estimating the prognosis of patients with head and neck squamous cell carcinoma (HNSCC) undergoing definitive treatment.

**Methods:**

To evaluate the prognostic potential of immune biomarkers, we conducted a prospective monocentric cohort study with loco-regionally advanced HNSCC patients indicated for definitive radiotherapy/radiochemotherapy at the Department of Oncology, Ostrava University Hospital, Czech Republic, between June 2020 and August 2023. We focused on the expression of programmed death ligand 1 (PD-L1) and tumor-infiltrating lymphocytes (TILs) relative to overall survival (OS) and specific survival rates. Associations between biomarkers and survival rates were assessed by crude and adjusted hazard ratios (cHR, aHR, respectively) obtained from Cox proportional hazards regression.

**Results:**

Among a total of 55 patients within a median follow-up of 19.7 months, there were 21 (38.2%) all-cause deaths and 15 (27.3%) cancer-related deaths. An overall survival (OS) rate of 61.8% and a disease-specific survival (DSS) rate of 72.7% were recorded. A significant association between survival rates and a ≥10% difference in PD-L1 expression on immune versus tumor cells (high PD-L1_IC_ expression) was documented regardless of the type of analysis (univariate or multivariate). In addition, a stronger association was confirmed for OS and the composite biomarker high PD-L1_IC_ expression along with either median-higher CD8+ TIL count or increased TIL density ≥30%, as indicated by an aHR of 0.08 (95% CI, 0.01 to 0.52) and 0.07 (95% CI, 0.01 to 0.46), respectively. Similar results were demonstrated for other specific survival rates.

**Discussion:**

The early outcomes of the present study suggest the utility of a strong prognostic factor involving a composite biomarker high PD-L1_IC_ expression along with increased TIL density in HNSCC patients undergoing definitive radiotherapy and radiochemotherapy.

**Trial registration:**

The study is registered with Clinicaltrials.gov. – NCT05941676

## 1 Introduction

Estimation of the prognosis of patients with head and neck squamous cell carcinoma (HNSCC) has become increasingly important in recent years. Particularly in the curative stages of the disease, an accurate assessment of a patient’s prognosis can help optimize the treatment strategy and thus achieve a better balance between the ultimate therapeutic goal versus acceptable side effects and treatment toxicity. Therefore, the search for new prognostic markers is highly desirable. It is in this respect that research in the field of immuno-oncology offers new insights. Understanding the concept of the tumor immune microenvironment (TIME) and studying interactions between tumor cells (TCs) and immune cells (ICs) allows for new research perspectives including the search for prognostic immune biomarkers (1, 2). Of particular interest is the study of signaling inhibitory molecules expressed on the surface of both TCs and ICs that contribute to and modify these complex immune interactions (3, 4). This is especially important in HNSCC, which is considered a tumor with high immunogenic potential (5). The first biomarkers studied in TIME for their prognostic potential were tumor-infiltrating lymphocytes (TILs) (6, 7). Several studies have demonstrated an association between high TIL infiltration and favorable treatment outcomes in HNSCC patients (8, 9). Among the different subtypes of TILs, CD8+ T-lymphocytes have been shown to have the dominant prognostic potential (10). Furthermore, these findings have been summarized and comprehensively analyzed in meta-analyses (11, 12).

In addition to TILs, an important role in TIME is played by a group of immunoreactive molecules that modulate the complex immune interactions. These molecules represent signaling molecules expressed on TCs and ICs that generally have immunosuppressive activity. The most studied molecule is programmed death ligand 1 (PD-L1).

Its main activity is interaction with the programmed death receptor-1(PD-1) located on CD8+ T-lymphocytes, leading to cell inactivation and immunosuppression (13). Although the mechanism of PD-L1/PD-1 interaction is well understood, the prognostic potential of PD-L1 expression in HNSCC remains unclear.

Several studies have evaluated the association between PD-L1 expression and survival in HNSCC patients undergoing definitive treatment. The results either showed no effect of the biomarker or were associated with worse treatment outcomes (14–16). In addition, two previous meta-analyses did not demonstrate a prognostic effect, while another meta-analysis found PD-L1 expression to be a marker of poor prognosis in an Asian population (17–19). Furthermore, several studies have not demonstrated the feasibility of using the combined positive score (CPS) in PD-L1 assessment to estimate the prognosis of HNSCC patients (20–22). However, another three studies reported that PD-L1 expression appears to have a prognostic potential, particularly when assessing its expression on immune cells (23–25). A recent meta-analysis reported similar results (26).

Given the current findings, we designed a prospective cohort study to assess the association of survival rates and biomarkers, including PD-L1 expression and TIL density, in HNSCC patients indicated for definitive radiotherapy and/or radiochemotherapy. The early outcomes of our study focused not only on the individual biomarkers but, also, on their combination. Moreover, we attempted to define the cutoff value for higher or increased levels of biomarkers suitable for everyday clinical practice.

## 2 Materials and methods

### 2.1 Study design and participants

The ONKOL-01-Head and Neck Study was conducted as a monocentric prospective cohort study at the Department of Oncology of Ostrava University Hospital between June 2020 and August 2023.

The study aimed to assess prognostic factors for patients with head and neck squamous cell carcinoma (HNSCC) including human papillomavirus (HPV)-positive carcinomas indicated for definitive radiotherapy and/or radiochemotherapy. The inclusion criteria encompassed adults aged 18 to 90 with histologically verified HNSCC in the oral cavity, nasal cavity, oropharynx, larynx, or hypopharynx.

Patients diagnosed with non-metastatic clinical stages of disease (I-IVb) were eligible for participation provided their paraffin-embedded tumor tissue samples from the biopsy were available.

Patients with histological types other than HNSCC (such as nasopharyngeal, salivary gland, thyroid, mucosal melanoma, skin carcinomas, lymphomas, and occult primary tumors), as well as those with distant metastases, synchronous malignancies, recurrent disease or a history of prior radiotherapy or chemotherapy, were excluded. The initial diagnostic protocol included a comprehensive otorhinolaryngological examination. Computed tomography (CT) and/or magnetic resonance imaging (MRI) of the neck was conducted to determine the disease stage, which followed the American Joint Committee on Cancer TNM staging system, version 8 (27).

Prior to participation, informed consent was obtained from each patient. The study adhered to the ethical guidelines outlined in the Declaration of Helsinki and received approval from the Ethics Committee of Ostrava University Hospital.

### 2.2 Treatment and follow-up

Patients in the study were selected for definitive radiotherapy and/or radiochemotherapy. The intensity-modulated radiotherapy (IMRT) technique, utilizing 4-MV and 6-MV energy X-rays, was employed. The standard radiotherapy protocol comprised 70 Gy over 7 weeks, with a daily dose of 2.0 Gy/fraction, either as a stand-alone treatment or in combination with cisplatin chemotherapy.

Concurrent chemotherapy was administered on a weekly or triweekly basis, following guideline recommendations, with cumulative doses exceeding 200 mg/m² (28). Patients deemed unsuitable for chemoradiation were directed towards either hyperfractionated RT or accelerated RT (29, 30). Within a subgroup of patients with advanced oral cavity tumors, a dose escalation regimen employing CyberKnife stereotactic radiotherapy (SRT) was implemented (31).

The post-treatment follow-up protocol encompassed otorhinolaryngology and/or orofacial surgery examinations, endoscopic evaluations, laboratory tests including SCC-antigen tumor markers, positron emission tomography (PET/CT) as well as CT and/or MRI scans. Patients had follow-up appointments every 1 to 3 months during the initial 2 years, followed by intervals of 3 to 6 months thereafter. PET/CT scans, utilized to assess tumor response, were scheduled 12 weeks after treatment completion, with a subsequent scan after another 12 weeks. In instances of complete remission, CT or MRI scans were scheduled at 3- to 6-month intervals. In cases where disease progression or recurrence was evident, a combination of repeat examinations (PET/CT or CT and MRI) alongside endoscopic and/or invasive diagnostic procedures was performed. Information on the follow-up schedule is provided in **Supplementary Table S1**.

### 2.3 Immunohistochemical analysis

Immunohistochemical (IHC) staining was employed to assess immune biomarkers in pretreatment tissue samples that were formalin-fixed and paraffin-embedded. Tumor-infiltrating lymphocytes (TIL) were categorized based on the surface expression of CD3, CD4, and CD8. The expression of PD-L1 was evaluated on the membranes of both tumor and immune cells. Sections measuring 4 µm in thickness were extracted from representative paraffin blocks onto electrostatic glass slides. Immunohistochemistry was conducted using a customized protocol on a Ventana Benchmark Ultra instrument ^(Roche Diagnostics,Switzerland)^ employing an ultraView Universal DAB Detection Kit ^(Roche Diagnostics,Switzerland)^ after prior revitalization in CC1 buffer.

Specific antibodies were utilized for the detection of particular markers: PD-L1 (clone 22C3, DAKO M3653, dilution 1:50, incubation 40 minutes), CD3 (clone LN10, Leica NCL-LCD3-565, dilution 1:100, incubation 60 minutes), CD4 (clone SP35, DCS CI851C003, dilution 1:50, incubation 32 minutes), CD8 (clone P17-V, DB Biotech M0755, dilution 1:200, incubation 60 minutes). The cytokeratin marker (CK) was detected with clone AE1/AE3, Zytomed MSK019 (dilution 1:200, incubation 24 min) and was used to differentiate the epithelial component of the tumor parenchyma from the stroma.

Histopathological analysis was performed using an Olympus BX45 optical microscope^(Boston Industries Inc.,Boston)^, aided by an optical measuring grid allowing the assessment of 1 mm² under observation at a total magnification of 100× (objective 10×, eyepiece 10×). As part of the immnohistochemical analysis, the count of positive elements per 1 mm² within tumor tissue (parenchyma) was conducted for CD3, CD4, and CD8 antibodies. Median values were employed as TIL cutoff points to distinguish high versus low infiltration.

Moreover, TIL density within the examined tumor region was semiquantitatively assessed by calculating the proportion of area occupied by mononuclear (CD3+) cell infiltrates to the entire parenchymal area (% TIL area occupied by mononuclear cells in tumor parenchyma).

This assessment classified TIL densities as either decreased (<30%) or increased (≥30%) infiltration (32, 33), PD-L1 expression was individually evaluated in tumor parenchyma for both tumor cells (TCs) and immune cells (ICs), with positivity >1% indicated by the percentage intensity of ligand expression (34). Immune cells included intraparenchymal T-lymphocytes, macrophages, and dendritic cells that infiltrated tumor cell nests. In addition, PD-L1 expression on immune cells was assessed by the percentage difference in expression between ICs and TCs. A difference of ≥10% was categorized as high PD-L1 expression on immune cells (PD-L1_IC_).

### 2.4 Statistical analysis

The study aimed to investigate the potential association between the survival of HNSCC patients and PD-L1 expression on both TCs and ICs, taking into account the presence of TIL infiltration. The primary endpoint was overall survival (OS), calculated from the commencement of radiotherapy to the date of death from any cause or the last follow-up visit. Secondary endpoints included disease-specific survival (DSS), locoregional-free survival (LRFS), disease-free survival (DFS), and distant metastatic-free survival (DMFS).

Disease-specific survival was censored from the initiation of radiotherapy to the date of cancer-related death or the last follow-up visit. Patients dying from causes unrelated to HNSCC were censored at their time of death. Locoregional-free survival was defined by radiological and/or pathological evidence of tumor recurrence or progression at the primary site or in the regional nodal area detected after the initiation of radiotherapy. The parameters DFS and DMFS were used to record any form of recurrence or the detection of distant tumor metastasis, measured from the initiation of radiotherapy to the date of recurrence detection. Patients without tumor recurrence were censored at the time of their last follow-up contact.

Categorical demographic and disease variables were presented as proportions, while continuous variables were transformed into binary form based on the median values within the study population. Categorical variables underwent assessment using Fisher’s exact test or the χ² test. Cox proportional hazards regression was employed for both univariate and multivariate analyses, with adjustments made for covariates including sex, age, alcohol abuse, smoking status, HPV positivity, stage, concomitant chemotherapy, COVID vaccination status, and bacterial infection. The outcomes were expressed by crude (cHR) or adjusted hazard ratio (aHR). The power of the test was estimated based on a binary covariate in a Cox proportional hazards model considering the hazard ratio, sample size, overall probability of failure, and a default significance level α = 0.05.

All tests were two-tailed, with a level of significance set at 0.05. Statistical tests and analyses were conducted using Prism 9 (GraphPad Software, Inc., San Diego, CA, USA) and STATA version 17 software (StataCorp, College Station, TX, USA).

### 2.5 Resource identification initiative

Catalog numbers and RRID:

PDL-1 [clone 22C3, DAKO M3653 (Agilent Cat# M3653, RRID : AB_2861298)].
CD3 [clone LN10, Leica NCL-LCD3-565, (Leica Biosystems Cat# NCL-L-CD3-565 (also CD3-565-L-CE, RRID : AB_563541)].
CD4 [clone SP35, DCS CI851C003, (Thermo Fisher Scientific Cat# MA5-16338, RRID : AB_2537857)].
CD8 [clone P17-V, DB Biotech M0755, (DB Biotech Cat# DB 085, RRID : AB_2315705)].
CK [clone AE1/AE3, Zytomed MSK019, (Zytomed Systems Cat# MSG019 (also BMS006, MSK019-05, MSK019), RRID : AB_2864453)].

## 3 Results

A total of 55 patients diagnosed with localized and locoregionally advanced HNSCC, who were candidates for definitive radiotherapy and/or radiochemotherapy, were enrolled in the study from June 1, 2020, to August 9, 2022. The median age was 63 years (IQR: 58–71), with a higher proportion of male patients, 41 (74.5%). The predominant primary tumor sites included the oral cavity, 21 (38.2%) and oropharynx, 21 (38.2%), followed by hypopharynx, 7 (12.7%), and larynx, 6 (10.9%). Among the 21 oropharyngeal carcinomas, 15 (71.4%) were HPV-positive. Stage IV was the most common, represented by 37 (67.2%) patients, followed by stage III in 17 (31.0%) patients including one patient in stage I. The detailed clinicopathological characteristics of the cohort and tumor and treatment characteristics are provided in **Table 1** and **Supplementary Table S2**. The median follow-up time for overall survival (OS) and disease-specific survival (DSS) was 19.7 months (IQR: 10.1–25.1 months). Other specific survival rates were followed for shorter durations, ranging from 11.8 to 15.2 months.

**Table 1:** Patient and tumor characteristics.

Characteristic		No. of patients (%)	p
**Sex**	Female	14 (25.5)	<0.0001
	Male	41 (74.5)	
**Age**	< M	27 (49)	n.s.
	≥ M	28 (51)	
**CCI**	< M	27 (49)	n.s.
	≥ M	28 (51)	
**BMI**	< M	27 (49)	n.s.
	≥ M	28 (51)	
**Alcohol abuse**	No	25 (45.5)	n.s.
	Yes	30 (54.5)	
**Smoking**	No	11 (20)	<0.0001
	Yes*	44 (80)	
**Bacterial infection**	No	12 (21.8)	<0.0001
	Yes	43 (78.2)	
**COVID vaccination**	No	23 (41.8)	n.s.
	Yes	32 (58.2)	
**Tumor site**	Oropharynx	21 (38.2)	0.0001
	Oral cavity	21 (38.2)	
	Hypopharynx	7 (12.7)	
	Larynx	6 (10.9)	
**Stage of disease**	≤3**	18 (32.7)	0.0003
	4	37 (67.3)	
**Tumor grade**	1	21 (38.2)	0.0117
	2	24 (43.6)	
	3	10 (18.2)	
**HPV positivity**	No	40 (72.7)	<0.0001
	Yes	15 (27.3)	
**Concomitant CHT**	No	25 (45.5)	n.s.
	Yes	30 (54.5)	
**BED_10_**	< M	38 (69)	<0.0001
	≥ M	17 (31)	

*) Regular or former smoker; **) Only one patient in stage 1; M = median (age = 63 years; CCI = 5; BMI = 23.6 kg/m^2^; BED_10 = _80 Gy); CCI, Charlson Comorbidity Index; BMI, Body Mass Index; COVID, Coronavirus Disease; HPV, human papillomavirus; CHT, chemotherapy; BED_10_, Biological Effective Dose; p, p-value; n.s., not significant.

**Figure 1:**
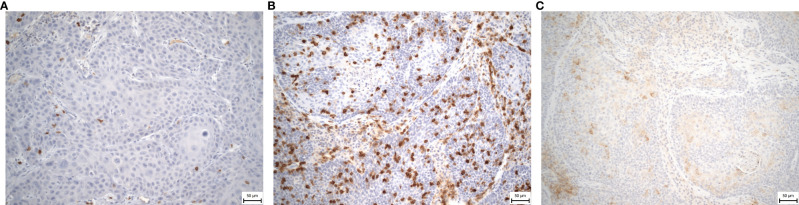
**(A–C)** Representative immunohistochemical staining sample of tumor tissue sample from HNSCC patients showing **(A)** low density of CD3+ TIL, original magnification x200; **(B)** elevated density of CD3+ TIL, original magnification x200; **(C)** High PD-L1_IC_ expression, original magnification x200; HNSCC, head and neck squamous cell carcinoma; TIL, tumor-infiltrating lymphocytes; PD-L1 – programmed death ligand 1; IC – immune cells.

Immunohistochemical analyses of pretreatment tumor tissue samples for the presence of TIL and PD-L1 expression were performed in 52 and 53 patients, respectively. In the remaining patients, the analysis could not be performed due to damage and degradation of the tissue sample. The median CD8+ TIL and CD4+ TIL counts were 28 and 32/mm^2^, respectively. Out of the 53 patient tissue samples evaluated for PD-L1 expression, it was present in 22 (41.5%) patients on tumor cells, and expression was higher than the median of 15% on immune cells in 52 (98%) patients. A TIL density of ≥30% assessed as increased infiltration was observed in 28 (53.8%) patients (**Figures 1A, B**). Moreover, high PD-L1_IC_ expression was documented in 20 (37.7%) patients (**Figure 1C**).

A total of 21 (38.2%) patient deaths during a median follow-up of 19.7 months accounted for 61.8% of OS and 72.7% of DSS. Out of the 55 patients, 3 (5.5%) had local recurrence, 13 (23.6%) had locoregional recurrence, 10 (18.2%) patients experienced distant metastatic progression and 15 (27.3%) patients died of cancer.

Univariate analysis did not show any association of overall or specific survivals with demographic and standard clinicopathological disease characteristics (**Supplementary Table S3**). Moreover, patient survival rates were not linked to the use of concomitant chemotherapy or various radiotherapy regimens and the biologically effective doses delivered.

The association between the OS rate and PD-L1 expression on ICs, CD8+ TIL infiltration, or increased TIL density, revealed through univariate analysis, was not confirmed by multivariate analysis, except for the relationship between OS and CD8+ TIL (**Table 2**; **Supplementary Table S4**). Conversely, specific DFS and DSS rates were shortened in patients with PD-L1 expression on TCs, as demonstrated by the respective aHR or cHR. Single factor-related survival rates were demonstrated for high PD-L1_IC_ expression - except for DMFS - regardless of type of analysis (univariate or multivariate).

**Table 2:** Association of single prognostic factors and outcome survivals, multivariate analysis.

			OS			DSS			DFS		
Prognostic factors		Total patients	No. of patients	aHR (95% CI)	p	No. of patients	aHR (95% CI)	p	No. of patients	aHR (95% CI)	p
**PD-L1 on TCs**	**Negative**	31	12	ref		7	ref		9	ref	
	**Positive**	22	9	1.49 (0.54–4.13)	n.s.	8	3.92 (1.04–14.75)	**0.043**	13	3.88 (1.42–10.59)	**0.008**
**PD-L1 on ICs**	**<M**	26	14	ref		9	ref		13	ref	
	**≥M**	27	7	0.44 (0.13–1.46)	n.s.	6	0.83 (0.19–3.66)	n.s.	9	0.90 (0.27–2.96)	n.s.
**PD-L1_IC_**	**Low**	33	17	ref		12	ref		18	ref	
	**High**	20	4	0.17 (0.04–0.74)	**0.018**	3	0.17 (0.03–0.92)	**0.039**	4	0.22 (0.05–0.92)	**0.038**
**CD4+ TILs**	**<M**	25	14	ref		11	ref		14	ref	
	**≥M**	27	7	0.62 (0.19–1.95)	n.s.	4	0.44 (0.10–1.86)	n.s.	8	0.71 (0.22–2.30)	n.s.
**CD8+ TIL s**	**<M**	25	15	ref		11	ref		14	ref	
	**≥M**	27	6	0.32 (0.11–0.95)	**0.041**	4	0.31 (0.08–1.18)	n.s.	8	0.63 (0.22–1.82)	n.s.
**TIL density**	**Decreased**	24	14	ref		10	ref		13	ref	
	**Increased**	28	7	0.38 (0.13–1.14)	n.s.	5	0.36 (0.09–1.40)	n.s.	9	0.51 (0.18–1.49)	n.s.

aHR, adjusted hazard ratio (adjustment for the covariates of sex, age, alcohol abuse, smoking status, HPV positivity, stage, concomitant chemotherapy, COVID vaccination status, and bacterial infection); CI, confidence interval; ref, reference; OS, Overall Survival; DSS, Disease-Specific Survival; DFS, Disease-Free Survival; p, p-value; M, median (15% for PD-L1 on ICs; 32/mm^2^ for CD4+ TIL; 28/mm^2^ for CD8+ TIL); TCs, tumor cells; Ics, immune cells; TIL, tumor-infiltrating lymphocytes; TIL density, Decreased <30%, Increased ≥ 30%; PD-L1, programmed death ligand 1; PD-L1_IC_, percentage difference between PD-L1 expression of immune cells and tumor cells - Low <10%, High ≥ 10%; n.s., not significant. Significant results are highlighted in bold.

**Figure 2:**
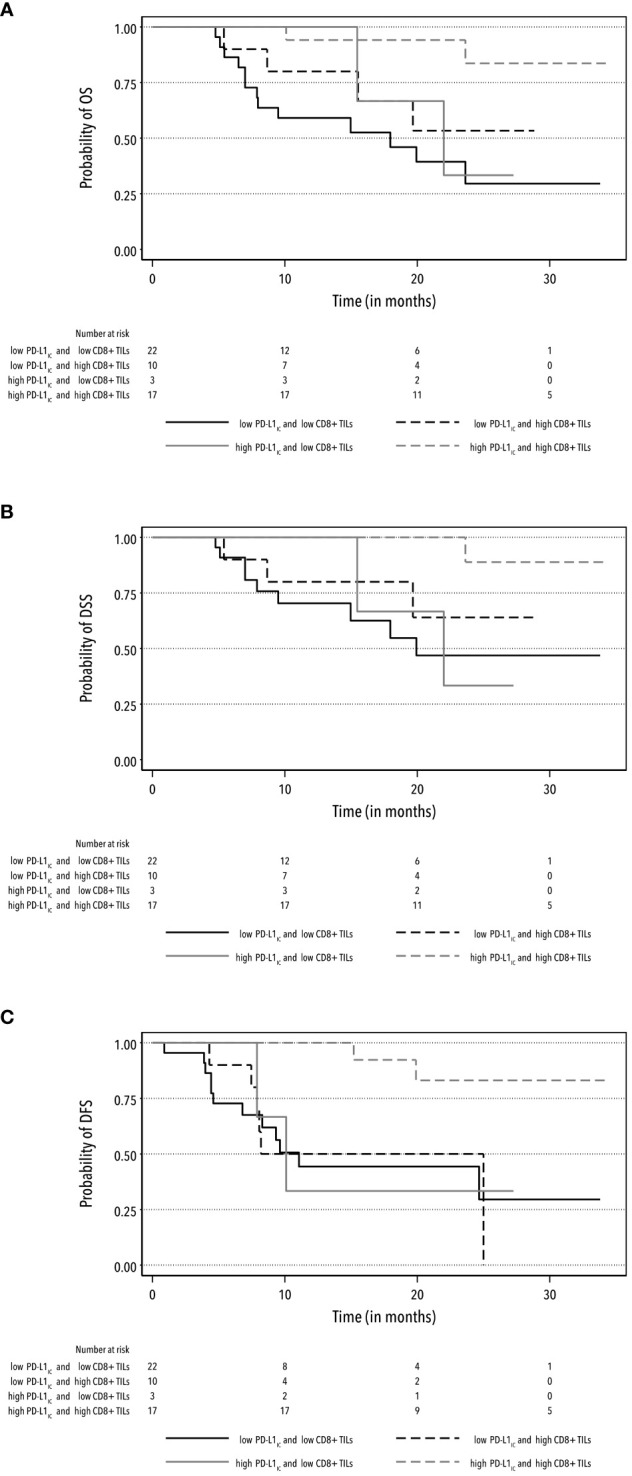
Kaplan-Meier curves for the rates of OS **(A)**, DSS **(B)** and DFS **(C)** according to the combination of high and low PD-L1_IC_ expression and high and low CD8+ TIL infiltration.

**Figure 3:**
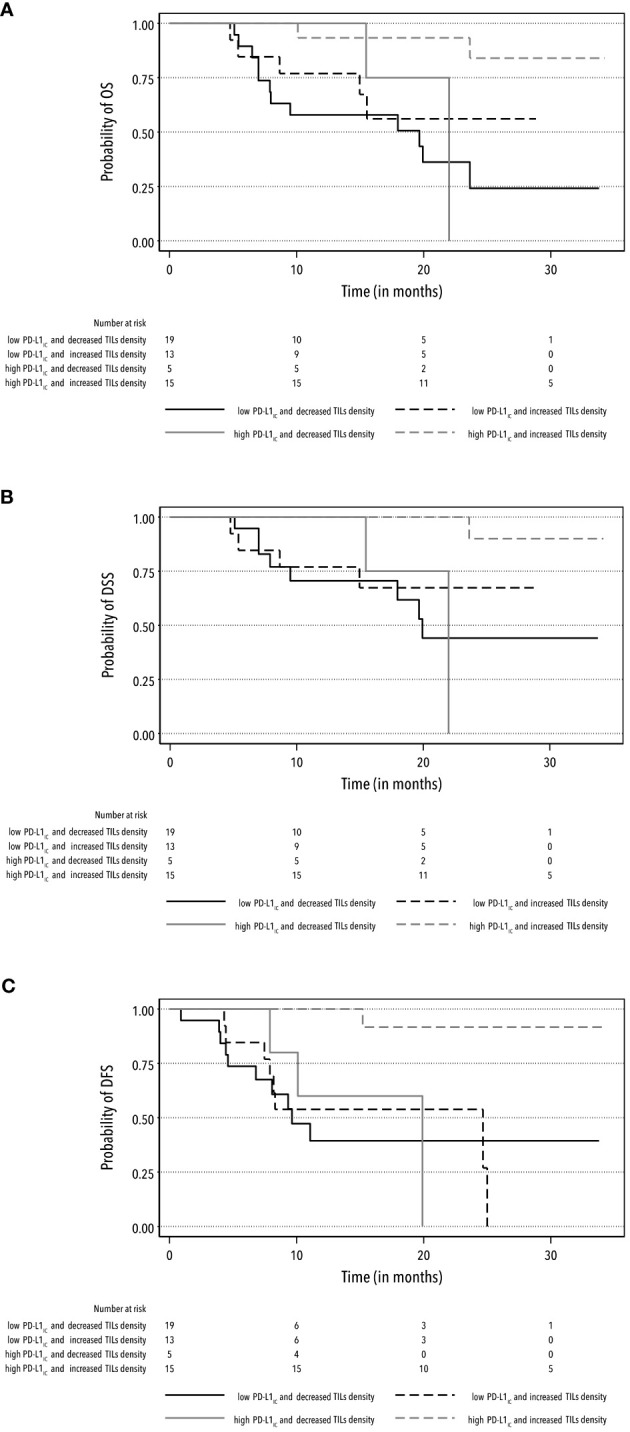
Kaplan-Meier curves for the rates of OS **(A)**, DSS **(B)** and DFS **(C)** according to the combination of high and low PD-L1_IC_ expression and increased and decreased TIL density.

The interaction of high PD-L1_IC_ expression, either with median-higher CD8+ TIL infiltration or increased TIL density, exhibited a similar prolonged effect on survival rates. The aHR for high PD-L1_IC_ expression and higher CD8+ TIL infiltration was 0.08 (95% CI: 0.01–0.52) for OS and 0.05 (95% CI: 0.00–0.61) for DSS. Similarly, the aHR for high PD-L1_IC_ expression and increased TIL density achieved 0.07 (95% CI: 0.01–0.46) for OS and 0.05 (95% CI: 0.00–0.61) for DSS with the power test of > 90% (**Supplementary Table S5**). The outcomes of univariate and multivariate analyses for other specific survival rates are reported in **Table 3** (**Supplementary Table S6**). Furthermore, these findings were supported by Kaplan-Meier curves for OS, DSS, and DFS survival rates (**Figures 2**, **3**).

**Table 3:** Association of interaction of prognostic factors and outcome survivals, multivariate analysis.

			OS			DSS			DFS		
Prognostic factor (1)	Prognostic factor (2)	Total patients	No. of patients	aHR (95% CI)	p	No. of patients	aHR (95% CI)	p	No. of patients	aHR (95% CI)	p
**PD-L1_IC_**	**CD8+ TILs**										
**Low**	**<M**	22	13	ref		9	ref		12	ref	
**Low**	**≥M**	10	4	0.57 (0.15–2.13)	n.s.	3	0.61 (0.13–2.81)	n.s.	6	1.39 (0.42–4.65)	n.s.
**High**	**<M**	3	2	0.37 (0.05–2.62)	n.s.	2	0.40 (0.05–3.42)	n.s.	2	0.98 (0.12–8.36)	n.s.
**High**	**≥M**	17	2	0.08 (0.01–0.52)	**0.008**	1	0.05 (0.00–0.62)	**0.020**	2	0.15 (0.02–1.00)	**0.050**
**PD-L1_IC_**	**TIL density**										
**Low**	**Decreased**	19	12	ref		8	ref		10	ref	
**Low**	**Increased**	13	5	0.52 (0.16–1.66)	n.s.	4	0.71 (0.17–2.91)	n.s.	8	0.97 (0.33–2.83)	n.s.
**High**	**Decreased**	5	2	0.31 (0.04–2.18)	n.s.	2	0.70 (0.06–7.60)	n.s.	3	1.58 (0.25–10.18)	n.s.
**High**	**Increased**	15	2	0.07 (0.01–0.46)	**0.006**	1	0.05 (0.00–0.61)	**0.020**	1	0.04 (0.00–0.51)	**0.013**

aHR, adjusted hazard ratio (adjustment for the covariates of sex, age, alcohol abuse, smoking status, HPV positivity, stage, concomitant chemotherapy, COVID vaccination status, and bacterial infection); CI, confidence interval; ref, reference; OS, Overall Survival; DSS, Disease-Specific Survival; DFS, Disease-Free Survival; p, p-value; M, median (28/mm^2^ for CD8+ TIL); TCs, tumor cells; TIL, tumor-infiltrating lymphocytes; TIL density, Decreased <30%, Increased ≥ 30%; PD-L1, programmed death ligand 1; PD-L1_IC_, percentage difference between PD-L1 expression of immune cells and tumor cells - Low <10%, High ≥ 10%; n.s., not significant. Significant results are highlighted in bold.

## 4 Discussion

This is a unique prospective study that aimed to assess the prognostic impact of immune biomarkers, including TIL and PD-L1 expression, alone or in combination, in HNSCC patients with loco-regionally advanced tumors undergoing definitive radiotherapy and/or radiochemotherapy. Both biomarkers demonstrated a potential prognostic effect on OS, DSS, and DFS, as supported by their respective hazard ratios. When evaluating the impact of a single biomarker, high CD8+ lymphocyte infiltration was associated with favorable outcomes in terms of OS, DFS, and DSS. Previous studies and meta-analyses have reported comparable results (11, 12). This effect may be explained by the activity of CD8+ lymphocytes in recognizing and destroying tumor cells, making them key elements of the anti-tumor immune response. Increased TIL density exhibited a prognostic effect on survival rates similar to high levels of CD8+ TIL as per the study median. The advantage of TIL density over CD8+ TIL consisted of the pre-defined cutoff value of elevated or high levels for each patient. In addition, TIL density takes into account other cell subtypes (CD4+, FOXP3) besides CD8+, thus providing a more comprehensive description of the conditions and immune interactions in the tumor microenvironment that contribute to the overall results. These conditions were extensively studied in preclinical research which provided a description of the complex modulation of the CD8+ anti-tumor response involving the immunosuppressive activity of CD4+ and FOXP3 lymphocytes (35). Thus, in the context of these findings, TIL density reflects the overall anti-tumor immune activity more accurately than a single immune biomarker (CD8+ lymphocyte level).

Conversely, no favorable effect of PD-L1 expression on ICs or TCs alone was observed, except for a shortened DSS and DFS in patients with high expression on TCs. Which corresponds to the primary pathophysiolgic mechanism of action of PD-L1 expression that cancer cells use to escape immune surveillance (36). As both PD-L1 expressions on ICs and TCs were determined in the same tissue section sample, we applied the criterion of higher expression on ICs compared to TCs, defined by a difference of ≥10%. The higher expression of PD-L1 on ICs increased the survival rates including OS, DSS, DFS, and LRFS, proving itself as a potentially new prognostic factor. Moreover, this approach eliminates the currently varying cutoff values of PD-L1 expression positivity (23–25).

In addition, the interaction of both the above biomarkers resulted in a strong prognostic effect. The survival rates of patients with high PD-L1_IC_ expression, along with median-higher CD8+ infiltration or increased TIL density, were significantly prolonged compared to those not meeting both criteria. This effect was evident in all investigated survival rates except for DMFS. This specific survival rate was likely insufficiently supported by the low number of patients with metastatic findings within our study follow-up.

Furthermore, a heightened PD-L1 expression on ICs relative to TCs, along with increased TIL density, is likely to play a role in modulating the complex immune interactions, thereby enhancing the T-cell-mediated antitumor effect. These are complex mechanisms, but probably involve the activity of antigen-presenting cells that stimulate the CD8+ cytotoxic response by inhibiting CD4+ and FOXP3 activity through PD-L1 expression (35). From this perspective, our findings suggest that the prognostic potential of immune biomarkers should only be assessed with careful consideration of their potential integration within the complex immune interactions to avoid drawing erroneous conclusions.

The most important finding of the present study is that the interaction of both the above biomarkers consistently shown to be strong prognostic factors independent of standard clinicopathological characteristics (sex, age, tumor stage, grade, HPV positivity), smoking history, alcohol abuse, radiotherapy regimen, or use of concomitant chemotherapy.The fact that no association between disease stage and survival rates was identified is likely due to the majority of patients being in advanced stages III or IV. Furthermore, our findings were consistent with the outcomes of previous studies demonstrating an association between PD-L1 expression on ICs and favorable OS (23–25). Nevertheless, none of those studies evaluated biomarker interactions; hence, their outcomes suggested slightly shorter survival rates compared to ours.

The limitations of the present study include the small number of patients enrolled and follow-up duration. We do not believe that the sample size could have influenced the final result, as the estimated power of the test exceeded 90% and, consequently, we anticipate the reproducibility of our findings. Since the median follow-up time was approximately two years, it remains uncertain whether additional follow-up time could affect these early survival outcomes. We believe that our proposed predictors could remain stable over a longer follow-up period even if their prognostic potential might decrease over time.

## 5 Conclusion

The outcomes of this study support the use of high PD-L1 expression on ICs in conjunction with increased TIL density as prognostic markers of the survival rates of HNSCC patients undergoing definitive radiotherapy and/or radiochemotherapy. Moreover, the applied definition of high expression of PD-L1_IC_ and increased TIL density could be implemented in common clinical practice. In future studies, it may be interesting to explore different combinations of biomarkers, including PD-1, based on the patterns observed in our study.

## Data availability statement

The original contributions presented in the study are included in the article/**Supplementary Material**. Further inquiries can be directed to the corresponding author.

## Ethics statement

The studies involving humans were approved by The Ethics Committee of University Hospital Ostrava (protocol code 829/2020). The studies were conducted in accordance with the local legislation and institutional requirements. The participants provided their written informed consent to participate in this study. Written informed consent was obtained from the individual(s) for the publication of any potentially identifiable images or data included in this article.

## Author contributions

TB: Conceptualization, Data curation, Methodology, Project administration, Resources, Validation, Writing – original draft. MP: Conceptualization, Formal analysis, Methodology, Writing – original draft. PH: Conceptualization, Data curation, Formal analysis, Methodology, Writing – review & editing. PM: Investigation, Writing – review & editing. LK: Investigation, Writing – review & editing. ZC: Investigation, Writing – review & editing. JŠ: Funding acquisition, Investigation, Writing – review & editing. JC: Project administration, Writing – review & editing. RS: Conceptualization, Supervision, Writing – review & editing.
