# Supplementary material for: High PD-L1 expression on immune cells along with increased density of tumor-infiltrating lymphocytes predicts a favorable survival outcome for patients with loco-regionally advanced head and neck cancer: early results from a prospective study
Source: Front Oncol. 2024 Apr 4;14:1346793. doi: 10.3389/fonc.2024.1346793 (PMC11024328; doi:10.3389/fonc.2024.1346793)
Supplement: Supplementary file 1 [file Table_1.docx]

Supplementary Material

**Table S1. Follow-up schedule**

| **Follow-up schedule** | **Week 3** | **Week 12** | **Week 24** | **Month 9** | **Month 12** | **Month 18** | **Month 24** | **Month 30** | **Month**  **36** |
| --- | --- | --- | --- | --- | --- | --- | --- | --- | --- |
| **ENT exam** | ● | ● | ● | ● | ● | ● | ● | ● | ● |
| **Blood test** | ● | ● | ● | ● | ● | ● | ● | ● | ● |
| **MRI** | - | ○ | ○ | ○ | ○ | ○ | ○ | ○ | ○ |
| **PET/CT** | - | ● | ● | - | - | - | - | - | - |
| **CT** | - | - | - | ● | ● | ● | ● | ● | ● |

● – obligatory examination; ○ – facultative examination; ENT – clinical and/or endoscopic examination by otolaryngologist or orofacial surgeon; Blood test – blood count, biochemistry, SCCA marker, thyroid hormones; MRI – Magnetic resonance imaging optional in selected cases where CT or PET/CT do not provide sufficient information; PET/CT – positron emission computed tomography during the first 6 months to assess the dynamics of tumor regression after treatment and to distinguish between post-treatment changes and viable residual tumor; CT – computed tomography, after 36 months of follow-up, imaging examination is indicated less frequently, depending on patient and tumor characteristics.

**Table S2: Tumor and treatment characteristic**

| **Characteristic** |  | **No. of patients (%)** |
| --- | --- | --- |
| **Tumor site specification** | |  |
| **Anatomic subsite** | Hypopharynx | 7 (12.7) |
|  | Tonsila | 12 (21.8) |
|  | Soft palate | 2 (3.6) |
|  | Base of tongue | 5 (9.1) |
|  | Oral tongue | 5 (9.1) |
|  | Floor of the mouth | 11 (20) |
|  | Alveolar ridge | 7 (12.7) |
|  | Glottis | 3 (5.5) |
|  | Supraglottis | 3 (5.5) |
| **RT regimens** | |  |
| **Normofractionated** | 70Gy in 2Gy/day for 7 weeks (total 35 fractions) + cisplatin | 25 (45.5) |
|  | 70Gy in 2Gy/day for 7 weeks (total 35 fractions) | 5 (9.1) |
| **Hyperfractionated** | 72.5Gy in 1.45Gy per fraction two times per day for 5 weeks (total 50 fractions) | 5 (9.1) |
| **Dose escalated** | 70Gy in 2Gy/day for 7 weeks (total 35 fractions) + SBRT boost of 10Gy in 2fractions * | 7 (12.7) |
|  | 72.5Gy in 1.45Gy per fraction two times per day for 5 weeks (total 50 fractions) + SBRT boost of 10Gy in 2 fractions * | 4 (7.3) |
| **Accelerated** | 55Gy in 2,75Gy/dya for 4 weeks (total 20 fractions) | 1 (1.8) |
|  | 60Gy in a split course of 30Gy in 3Gy per fraction + 30Gy in 2Gy per fraction for 5 weeks (total 25fractions) | 8 (14.5) |
| **Chemotherapy regimen** | |  |
| **Type and dose of chemotherapy** | Cisplatin 100mg/m2 on days 1, 22 and 43 | 13 (23.6) |
|  | Cisplatin 40mg/m2 on weeks 1-6 | 12 (21.8) |
|  | No chemotherapy | 30 (54.6) |
| **Cumulative dose of cisplatin > 200mg** | No | 9 (16.4) |
|  | Yes | 16 (29.1) |
|  | None | 30 (54.5) |

*) Only for patients with floor of the mouth tumors; RT = radiotherapy; SBRT = stereotactic body radiotherapy;

**Table S3: Survival rates in relationship to patients and tumor characteristics**

| **Parameter** | |  | **OS** | | | **DSS** | | | **DFS** | | | **DMFS** | | | **LRFS** | | | |
| --- | --- | --- | --- | --- | --- | --- | --- | --- | --- | --- | --- | --- | --- | --- | --- | --- | --- | --- |
|  |  | **Total patients** | **No patients** | **cHR** | **p** | **No patients** | **cHR** | **p** | **No patients** | **cHR** | **p** | **No patients** | **cHR** | **p** | **No patients** | **cHR** | **p** |  |
| **Sex** | **Female** | 14 | 4 | ref |  | 3 | ref |  | 7 | ref |  | 2 | ref |  | 5 | ref |  |  |
|  | **Male** | 41 | 17 | 1.92 (0.64-5.72) | 0.245 | 12 | 1.83 (0.51–6.54) | 0.351 | 15 | 0.79 (0.32–1.94) | 0.602 | 8 | 2.02 (0.42–9.71) | 0.382 | 11 | 0.80 (0.28–2.31) | 0.682 |  |
| **Age** | **<M** | 27 | 7 | ref |  | 4 | ref |  | 8 | ref |  | 4 | ref |  | 6 | ref |  |  |
|  | **≥M** | 28 | 14 | 2.27 (0.91–5.64) | 0.078 | 11 | 3.18 (1.01–10.03) | 0.049 | 14 | 1.85 (0.78–4.43) | 0.165 | 6 | 1.65 (0.46–5.86) | 0.439 | 10 | 1.86 (0.67–5.12) | 0.231 |  |
| **CCI** | **<M** | 27 | 8 | ref |  | 6 | ref |  | 11 | ref |  | 4 | ref |  | 10 | ref |  |  |
|  | **≥M** | 28 | 13 | 2.06 (0.85–5.00) | 0.108 | 9 | 1.92 (0.68–5.42) | 0.217 | 11 | 1.15 (0.50–2.66) | 0.739 | 6 | 2.05 (0.58–7.31) | 0.267 | 6 | 0.64 (0.23–1.77) | 0.390 |  |
| **BMI** | **<M** | 27 | 13 | ref |  | 9 | ref |  | 14 | ref |  | 6 | ref |  | 11 | ref |  |  |
|  | **≥M** | 28 | 8 | 0.55 (0.23–1.34) | 0.188 | 6 | 0.60 (0.21–1.69) | 0.334 | 8 | 0.43 (0.18–1.03) | 0.058 | 4 | 0.48 (0.13–1.73) | 0.261 | 5 | 0.38 (0.13–1.10) | 0.076 |  |
| **Alcohol abuse** | **No** | 25 | 7 | ref |  | 6 | ref |  | 8 | ref |  | 2 | ref |  | 7 | ref |  |  |
|  | **Yes** | 30 | 14 | 2.00 (0·81–4·96) | 0.135 | 9 | 1.49 (0.53–4.19) | 0.452 | 14 | 1.92 (0.79–4.63) | 0.148 | 8 | 5.13 (1.07–24.59) | 0.041 | 9 | 1.19 (0.44–3.21) | 0.727 |  |
| **Smoking** | **No** | 11 | 4 | ref |  | 3 | ref |  | 3 | ref |  | 1 | ref |  | 3 | ref |  |  |
|  | **Yes*** | 44 | 17 | 1.00 (0.33–2.96) | 0.994 | 12 | 0.93 (0.26–3.31) | 0.915 | 19 | 1.70 (0.50–5.76) | 0.392 | 9 | 2.24 (0.28–17.65) | 0.445 | 13 | 1.11 (0.32–3.91) | 0.868 |  |
| **Bacterial infection** | **No** | 12 | 1 | ref |  | 0 | ref |  | 1 | ref |  | 1 | ref |  | 0 | ref |  |  |
|  | **Yes** | 43 | 20 | 7.11 (0.95–53.13) | 0.056 | 15 | - |  | 21 | 9.15 (1.22–68.71) | 0.031 | 9 | 3.88 (0.48–31.17) | 0.202 | 16 | - |  |  |
| **Tumor site** | **HP** | 7 | 3 | ref |  | 2 | ref |  | 3 | ref |  | 2 | ref |  | 2 | ref |  |  |
|  | **OC** | 21 | 9 | 1.29 (0.35–4.81) | 0.700 | 6 | 1.34 (0.27–6.69) | 0.722 | 8 | 0.93 (0.25–3.53) | 0.916 | 3 | 0.60 (0.10–3.63) | 0.577 | 6 | 1.09 (0.22–5.42) | 0.915 |  |
|  | **OP** | 21 | 7 | 0.75 (0.19–2.91) | 0.676 | 5 | 0.83 (0.16–4.29) | 0.824 | 8 | 0.74 (0.19–2.78) | 0.651 | 3 | 0.36 (0.06–2.15) | 0.261 | 7 | 1.11 (0.23–5.33) | 0.900 |  |
|  | **L** | 6 | 2 | 1.04 (0.17–6.26) | 0.966 | 2 | 1.64 (0.23–11.77) | 0.623 | 3 | 1.06 (0.21–5.28) | 0.942 | 2 | 1.21 (0.17–8.63) | 0.852 | 1 | 0.54 (0.05–5.97) | 0.616 |  |
| **Stage of disease** | **≤3**** | 18 | 6 | ref |  | 5 | ref |  | 9 | ref |  | 6 | ref |  | 5 | ref |  |  |
|  | **4** | 37 | 15 | 1.56 (0.60–4.04) | 0.364 | 10 | 1.24 (0.42–3.67) | 0.692 | 13 | 0.86 (0.37–2.03) | 0.736 | 4 | 0.43 (0.12–1.52) | 0.187 | 11 | 1.24 (0.43–3.58) | 0.69 |  |
| **Tumor grade** | **1** | 21 | 9 | ref |  | 6 | ref |  | 8 | ref |  | 3 | ref |  | 7 | ref |  |  |
|  | **2** | 24 | 9 | 0.80 (0.32–2.01) | 0.631 | 7 | 0.92 (0.31–2.75) | 0.883 | 10 | 0.96 (0.38–2.44) | 0.930 | 4 | 1.00 (0.22–4.48) | 0.998 | 8 | 0.93 (0.33–2.56) | 0.881 |  |
|  | **3** | 10 | 3 | 0.66 (0.18–2.44) | 0.532 | 2 | 0.65 (0.13–3.23) | 0.600 | 4 | 0.93 (0.28–3.11) | 0.911 | 3 | 1.96 (0.39–9.77) | 0.411 | 1 | 0.25 (0.03–2.06) | 0.200 |  |
| **Metastatic lymph node** | **Absent** | 17 | 7 | ref |  | 5 | ref |  | 8 | ref |  | 5 | ref |  | 5 | ref |  |  |
|  | **Present** | 38 | 14 | 0.74 (0.30–1.83) | 0.510 | 10 | 0.74 (0.25–2.16) | 0.577 | 14 | 0.65 (0.27–1.57) | 0.341 | 5 | 0.30 (0.09–1.06) | 0.061 | 11 | 0.92 (0.32–2.64) | 0.871 |  |
| **HPV positivity** | **No** | 40 | 18 | ref |  | 12 | ref |  | 17 | ref |  | 7 | ref |  | 13 | ref |  |  |
|  | **Yes** | 15 | 3 | 0.31 (0.09–1.04) | 0.058 | 3 | 0.46 (0.13–1.64) | 0.233 | 5 | 0.53 (0.20–1.45) | 0.219 | 3 | 0.69 (0.18–2.69) | 0.590 | 3 | 0.46 (0.13–1.62) | 0.229 |  |
| **CHT** | **No** | 25 | 12 | ref |  | 8 | ref |  | 11 | ref |  | 4 | ref |  | 7 | ref |  |  |
|  | **Yes** | 30 | 9 | 0.43 (0.18–1.04) | 0.060 | 7 | 0.50 (0.18–1.39) | 0.185 | 11 | 0.61 (0.26–1.43) | 0.259 | 6 | 0.75 (0.21–2.75) | 0.669 | 9 | 0.92 (0.34–2.48) | 0.875 |  |
| **RT dose** | **≥M** | 17 | 8 | ref |  | 6 | ref |  | 7 | ref |  | 3 | ref |  | 4 | ref |  |  |
|  | **<M** | 38 | 13 | 0.55 (0.22–1.34) | 0.186 | 9 | 0.50 (0.18–1.42) | 0.194 | 15 | 0.74 (0.30–1.85) | 0.521 | 7 | 0.62 (0.15–2.50) | 0.503 | 12 | 1.28 (0.41–3.97) | 0.669 |  |

*) Regular or former smoker; **) Only one patient with stage 1; M = median (age = 63 years; CCI = 5; BMI = 23.6 kg/m^2^; BED_10_ = 80 Gy; CCI = Charlson Comorbidity Index; BMI = Body Mass Index (M=; GGT = Gamma Glutamyl-Transferase; RT = radiotherapy; COVID = Coronavirus Disease; HPV = Human Papilloma Virus; CHT= chemotherapy; BED_10_ = Biological Effective Dose; CI = confidence interval; ref = reference; OS = Overall Survival; DSS = Disease-Specific Survival; DFS = Disease-Free Survival; DMFS = Distant Metastatic-Free Survival; LRFS = Locoregional-Free Survival; p = p-value; n.s. = not significant; HP = Hypopharynx; OC = Oral cavity; OP = Oropharynx; L = Larynx

**Table S4: Association of single prognostic factors and outcome survivals, univariate and multivariate analysis**

|  |  |  | **OS** |  |  | **DSS** |  |  | **DFS** |  |  | **DMFS** |  |  | **LRFS** |  |  |
| --- | --- | --- | --- | --- | --- | --- | --- | --- | --- | --- | --- | --- | --- | --- | --- | --- | --- |
| **Prognostic parameter** |  | **Total patients** | **No patients** | **cHR** | **aHR** | **No patients** | **cHR** | **aHR** | **No patients** | **cHR** | **aHR** | **No patients** | **cHR** | **aHR** | **No patients** | **cHR** | **aHR** |
| **PD-L1 on TCs** | **Negative** | 31 | 12 | ref | ref | 7 | ref | ref | 9 | ref | ref | 6 | ref | ref | 7 | ref | ref |
|  | **Positive** | 22 | 9 | 1.30 (0.55–3.09) | 1.49 (0.54–4.13) | 8 | 1.97 (0.71–5.43) | **3.92 (1.04–14.75)** | 13 | **2.9 (1.23–6.84)** | **3.88 (1.42–10.59)** | 4 | 1.15 (0.32–4.08) | 1.49 (0.31–7.25) | 9 | 2.27 (0.84–6.09) | 2.70 (0.80–9.13) |
| **PD-L1 on ICs** | **<M** | 26 | 14 | ref | ref | 9 | ref | ref | 13 | ref | ref | 6 | ref | ref | 10 | ref | ref |
|  | **≥M** | 27 | 7 | **0.38 (0.15–0.95)** | 0.44 (0.13–1.46) | 6 | 0.52 (0.18–1.45) | 0.83 (0.19–3.66) | 9 | 0.47 (0.20–1.11) | 0.90 (0.27–2.96) | 4 | 0.43 (0.12–1.53) | 0.28 (0.04–1.79) | 6 | 0.45 (0.16–1.25) | 1.34 (0.34–5.34) |
| **PD-L1_IC_** | **Low**  **(<10%)** | 33 | 17 | ref | ref | 12 | ref | ref | 18 | ref | ref | 8 | ref | ref | 13 | ref | ref |
|  | **High**  **(>10%)** | 20 | 4 | **0.26 (0.09–0.79)** | **0.17 (0.04–0.74)** | 3 | 0.28 (0.08–1.01) | **0.17 (0.03–0.92)** | 4 | **0.22 (0.07–0.64)** | **0.22 (0.05–0.92)** | 2 | 0.24 (0.05–1.16) | **0.11 (0.01–0.96)** | 3 | **0.26 (0.07–0.91)** | 0.44 (0.08–2.44) |
| **CD4+ TILs** | **<M** | 25 | 14 | ref | ref | 11 | ref | ref | 14 | ref | ref | 7 | ref | ref | 11 | ref | ref |
|  | **≥M** | 27 | 7 | 0.45 (0.18–1.11) | 0.62 (0.19–1.95) | 4 | 0.33 (0.10–1.03) | 0.44 (0.10–1.86) | 8 | **0.41 (0.17–0.99**) | 0.71 (0.22–2.30) | 3 | 0.30 (0.08–1.16) | 0.31 (0.04–2.26) | 5 | 0.37 (0.13–1.07) | 0.61 (0.15–2.43) |
| **CD8+ TIL s** | **<M** | 25 | 15 | ref | ref | 11 | ref | ref | 14 | ref | ref | 7 | ref | ref | 10 | ref | ref |
|  | **≥M** | 27 | 6 | **0.26 (0.10–0.68)** | **0.32 (0.11–0.95)** | 4 | **0.24 (0.08–0.76)** | 0.31 (0.08–1.18) | 8 | **0.35 (0.14–0.84)** | 0.63 (0.22–1.82) | 3 | **0.23 (0.06–0.88)** | 0.31 (0.05–1.80) | 6 | 0.42 (0.15–1.15) | 0.67 (0.19–2.36) |
| **TILs density** | **Decreased**  **(<30%)** | 24 | 14 | ref | ref | 10 | ref | ref | 13 | ref | ref | 5 | ref | ref | 12 | ref | ref |
|  | **Increased**  **(> 30%)** | 28 | 7 | **0.30 (0.12–0.76)** | 0.38 (0.13–1.14) | 5 | **0.30 (0.10–0.91)** | 0.36 (0.09–1.40) | 9 | **0.37 (0.16–0.89)** | 0.51 (0.18–1.49) | 5 | 0.46 (0.13–1.68) | 0.52 (0.10–2.67) | 4 | **0.21 (0.07–0.65)** | 0.27 (0.07–1.01) |

cHR = crude hazard ratio; aHR = adjusted hazard ratio (adjustment for the covariates of sex, age, alcohol abuse, smoking status, HPV positivity, stage, concomitant chemotherapy, COVID vaccination status, and bacterial infection); CI = confidence interval; ref = reference; OS = Overall Survival; DSS = Disease-Specific Survival; DFS = Disease-Free Survival; DMFS = Distant Metastatic-Free Survival; LRFS = Locoregional-Free Survival; p = p-value; M = median (15% for PDL1-IC; 32/mm^2^ for CD4+ TIL; 28/mm^2^ for CD8+ TILs); TCs = tumor cells; ICs = immune cells; TILs = tumor-infiltrating lymphocytes; PDL1 = programmed death ligand 1; PDL1_IC_ = percentage difference between PDL1 expression of immune cells and tumor cells; Significant results are highlighted in bold.

**Table S5: Power test for the combination of biomarker interactions in relation to OS**

| **PD-L1_IC_** | **TILs density** | **N** | **Event** | **Event probability** | **aHR (95%CI)** | **SE** | **SD** | **N sample size** | **Power of test** |
| --- | --- | --- | --- | --- | --- | --- | --- | --- | --- |
| **Low**  **(<10%)** | **Decreased**  **(<30%)** | 19 | 12 |  | Ref |  |  |  |  |
| **Low**  **(<10%)** | **Increased**  **(>30%)** | 13 | 5 | 0.53 | 0.52(0.16-1.66) | 0.31 | 1.75 | 32 | 27% |
| **High**  **(>10%)** | **Decreased**  **(<30%)** | 5 | 2 | 0.58 | 0.31(0.04-2.18) | 0.31 | 1.52 | 24 | 58% |
| **High**  **(>10%)** | **Increased**  **(>30%)** | 15 | 2 | 0.41 | **0.07(0.01-0.46)** | 0.07 | 0.41 | 34 | **99%** |

aHR = adjusted hazard ratio (adjustment for the covariates of sex, age, alcohol abuse, smoking status, HPV positivity, stage, concomitant chemotherapy, COVID vaccination status, and bacterial infection); OS = overall survival; CI = confidence interval; ref = reference; N= number;SE=standard error;SD=standard deviation; TILs = tumor-infiltrating lymphocytes; PD-L1 = programmed death ligand 1; PD-L1_IC_ = percentage difference between PD-L1 expression of immune cells and tumor cells;

**Table S6: Association of prognostic factors interaction and outcome survivals, univariate and multivariate analysis**

|  |  |  | **OS** |  |  | **DSS** |  |  | **DFS** |  |  | **DMFS** |  |  | **LRFS** |  |  |
| --- | --- | --- | --- | --- | --- | --- | --- | --- | --- | --- | --- | --- | --- | --- | --- | --- | --- |
| **Prognostic factor (1)** | **Prognostic factor (2)** | **Total patients** | **No patients** | **cHR** | **aHR** | **No patients** | **cHR** | **aHR** | **No patients** | **cHR** | **aHR** | **No patients** | **cHR** | **aHR** | **No patients** | **cHR** | **aHR** |
| **PD-L1_IC_** | **CD8+ TILs** |  |  |  |  |  |  |  |  |  |  |  |  |  |  |  |  |
| **Low**  **(<10%)** | **<M** | 22 | 13 | ref | ref | 9 | ref | ref | 12 | ref | ref | 6 | ref | ref | 8 | Ref | ref |
| **Low**  **(<10%)** | **≥M** | 10 | 4 | 0.53 (0.17–1.62) | 0.57 (0.15–2.13) | 3 | 0.58 (0.16–2.13) | 0.61 (0.13–2.81) | 6 | 0.98 (0.37–2.62) | 1.39 (0.42–4.65) | 2 | 0.44 (0.09–2.24) | 0.66 (0.07–6.32) | 5 | 1.32 (0.43–4.06) | 1.67 (0.40–6.91) |
| **High**  **(>10%)** | **<M** | 3 | 2 | 0.70 (0.16–3.10) | 0.37 (0.05–2.62) | 2 | 0.98 (0.21–4.58) | 0.40 (0.05–3.42) | 2 | 0.82 (0.18–3.70) | 0.98 (0.12–8.36) | 1 | 0.61 (0.07–5.09) | 0.23 (0.01–6.23) | 2 | 1.49 (0.32–7.03) | 2.58 (0.28–23.80) |
| **High**  **(>10%)** | **≥M** | 17 | 2 | **0.12 (0.03–0.53)** | **0.08 (0.01–0.52)** | 1 | **0.09 (0.01–0.69)** | **0.05 (0.00–0.62)** | 2 | **0.11 (0.02–0.50)** | **0.15 (0.02–1.00)** | 1 | **0.10 (0.01–0.83)** | **0.05 (0.00–1.00)** | 1 | **0.10 (0.01–0.81)** | 0.24 (0.02–2.91) |
| **PD-L1_IC_** | **TILs density** |  |  |  |  |  |  |  |  |  |  |  |  |  |  |  |  |
| **Low**  **(<10%)** | **Decreased**  **(<30%)** | 19 | 12 | ref | ref | 8 | ref | ref | 10 | ref | ref | 4 | ref | ref | 9 | ref | ref |
| **Low**  **(<10%)** | **Increased**  **≥30%** | 13 | 5 | 0.53 (0.18–1.50) | 0.52 (0.16–1.66) | 4 | 0.64 (0.19–2.14) | 0.71 (0.17–2.91) | 8 | 0.95 (0.37–2.41) | 0.97 (0.33–2.83) | 4 | 0.90 (0.22–3.66) | 0.77 (0.12–4.95) | 4 | 0.55 (0.16–1.81) | 0.75 (0.22–2.62) |
| **High**  **(>10%)** | **Decreased**  **<30%** | 5 | 2 | 0.53 (0.12–2.41) | 0.31 (0.04–2.18) | 2 | 0.80 (0.17–3.83) | 0.70 (0.06–7.60) | 3 | 0.85 (0.23–3.12) | 1.58 (0.25–10.18) | 1 | 0.62 (0.07–5.70) | 0.29 (0.01–7.75) | 3 | 0.93 (0.34–2.57) | 4.49 (0.65–30.91) |
| **High**  **(>10%)** | **Increased**  **(≥30%)** | 15 | 2 | **0.12 (0.03–0.54)** | **0.07 (0.01–0.46)** | 1 | **0.09 (0.01–0.72)** | **0.05 (0.00–0.61)** | 1 | **0.06 (0.01–0.44)** | **0.04 (0.00–0.51)** | 1 | 0.13 (0.01–1.17) | 0.05 (0.00–1.11) | 0 | 0.00 (0.00–0.00) | 0.00 (0.00–0.00) |

cHR = crude hazard ratio; aHR = adjusted hazard ratio (adjustment for the covariates of sex, age, alcohol abuse, smoking status, HPV positivity, stage, concomitant chemotherapy, COVID vaccination status, and bacterial infection); CI = confidence interval; ref = reference; OS = Overall Survival; DSS = Disease-Specific Survival; DFS = Disease-Free Survival; DMFS = Distant Metastatic-Free Survival; LRFS = Locoregional-Free Survival; p = p-value; M = median (28/mm^2^ for CD8+ TILs); TCs = tumor cells; TILs = tumor-infiltrating lymphocytes; PD-L1 = programmed death ligand 1; PD-L1_IC_ = percentage difference between PD-L1 expression of immune cells and tumor cells; Significant results are highlighted in bold.
